# A Potential Role for the Receptor for Advanced Glycation End-Products (RAGE) in the Development of Secondhand Smoke-Induced Chronic Sinusitis

**DOI:** 10.3390/cimb46010047

**Published:** 2024-01-13

**Authors:** Hannah Robin, Courtney Trudeau, Adam Robbins, Emily Chung, Erum Rahman, Olivia Gangmark-Strickland, Frank W. Licari, Duane R. Winden, Dan L. Orr, Juan A. Arroyo, Paul R. Reynolds

**Affiliations:** 1 College of Dental Medicine, Roseman University of Health Sciences, South Jordan, UT 84095, USA; 2 Oral & Maxillofacial Surgery, University Medical Center, Las Vegas, NV 89102, USA; 3 Lung and Placenta Laboratory, Department of Cell Biology and Physiology, Brigham Young University, Provo, UT 84602, USA

**Keywords:** inflammation, sinusitis, RAGE

## Abstract

Chronic sinusitis (CS) is characterized by sinonasal inflammation, mucus overproduction, and edematous mucosal tissue. CS impacts one in seven adults and estimates suggest up to 15% of the general U.S. population may be affected. This research sought to assess a potential role for receptors for advanced glycation end-products (RAGE), an inflammatory receptor expressed in tissues exposed to secondhand smoke (SHS). Human sinus tissue sections were stained for RAGE and S100s, common RAGE ligands. Wild-type mice and mice that over-express RAGE in sinonasal epithelium (RAGE TG) were maintained in room air (RA) or exposed to secondhand smoke (SHS) via a nose-only delivery system five days a week for 6 weeks. Mouse sections were stained for RAGE and tissue lysates were assayed for cleaved caspase 3, cytokines, or matrix metalloproteases. We discovered increased RAGE expression in sinus tissue following SHS exposure and in sinuses from RAGE TG mice in the absence of SHS. Cleaved caspase-3, cytokines (IL-1β, IL-3, and TNF-α), and MMPs (-9 and -13) were induced by SHS and in tissues from RAGE TG mice. These results expand the inflammatory role of RAGE signaling, a key axis in disease progression observed in smokers. In this relatively unexplored area, enhanced understanding of RAGE signaling during voluntary and involuntary smoking may help to elucidate potential therapeutic targets that may attenuate the progression of smoke-related CS.

## 1. Introduction

Sinonasal inflammation is observed in CS and is characterized by the presence of pro-inflammatory exudates, mucus overproduction, and elevated edematous mucosal tissue. According to the National Health Interview Survey of 1996, chronic sinusitis was the second most prevalent chronic health condition, affecting 12.5% of the US population or approximately 31 million patients each year [1,2]. More recently, it was determined that CS affects approximately one in seven adults and estimates presently suggest up to 15% of the general U.S. population may be sufferers [3,4,5]. Importantly, CS accounts for over 20% of all office visits to allergy and immunology specialists, and there are an estimated 18 million cases and 30 million courses of antibiotics delivered each year [6,7].

CS patients who have failed medical management may require surgical intervention in the form of nasal lavage, the creation of a nasoantral window, or endoscopic nasal surgery. The cost of this disease in the U.S. is estimated to be USD 6 billion dollars annually [4,5]. Although current therapies are effective in many patients, a substantial number of patients continue to have persistent sinus symptoms that reduce quality of life. The histologic changes in CS include damage to ciliated nasal epithelium, goblet cell hyperplasia and basement membrane denudation with an inflammatory component composed of Th2 lymphocytes, eosinophils, and mast cells [8]. In some patients with CS, nasal polyps may exist composed of edematous stroma, neutrophilic inflammation, hyperplasia of goblet cells, and squamous metaplasia [9].

Active smoking and SHS exposure is acknowledged as a key risk factor for the development of CS [10]. Several mechanisms of tobacco smoke-induced inflammation in CS have been proposed and include creation of reactive oxygen species (ROS) including superoxide, peroxide and hydroxyl radicals, persistent chronic bacterial or fungal infections, direct injury to the nasal epithelial barrier with resultant damage to ciliated epithelium, innate immunity dysfunction, and allergic factors [11,12]. All of these mechanisms most likely give rise to increased expression of pro-inflammatory cytokines. It is also essential to consider genetic mechanisms and the effects of cigarette smoke exposure and progression of CS. Gene products such as inflammatory cytokines, extracellular proteins, and elevated protease production (such as matrix metalloproteases) after prolonged cigarette smoke exposure are likely to stimulate the recruitment of inflammatory cells and histological changes observed in the nasal mucosa. The literature strongly suggests a relationship between SHS exposure and CS that warrants focused investigation [10,11,12].

While a number of mechanisms may cause CS, our attention has been directed to the possibility that SHS-induced RAGE and its ligands have a principal role in the inflammation and progression of CS. RAGE is a multi-ligand member of the immunoglobulin superfamily of cell-surface proteins and is expressed in many cell types including endothelial, vascular smooth muscle, fibroblasts, neurons, and macrophages/monocytes [13]. RAGE expression is most abundant in the lung where it primarily is selectively localized to the basolateral membranes of well-differentiated alveolar type I epithelial cells [14,15]. RAGE is upregulated wherever its ligands accumulate, and ligand binding with RAGE results in rapid and sustained cellular activation and gene transcription [14,16,17]. Its involvement in inflammation has been suggested by many studies, and it is upregulated in most inflammatory lesions studied to date [17,18,19,20,21,22,23,24,25]. Soluble RAGE (sRAGE), which is a truncated form of the receptor spanning the extracellular ligand-binding domain, and endogenous secreted RAGE (esRAGE) generated by alternative splicing, competes with full-length RAGE for ligand binding. These competitors may therefore reduce inflammatory responses, demonstrating potential success in the alleviation of inflammation [23,26,27].

Ligands for RAGE include advanced glycation end products (AGEs), HMGB1, and S100/calgranulins. As a pattern recognition receptor (PRR), RAGE recognizes the three-dimensional structure of ligands rather than specific amino acid sequences [26,28,29]. AGEs are products of nonenzymatic glycoxidation and oxidation of proteins and they are detected in tobacco smoke [30,31]. HMGB1 is a 219-amino acid protein that has two basic structural units termed HMGB1 box A and B, and a carboxy tail. HMGB1 may be released from necrotic cells, but not apoptotic cells, and is actively secreted by macrophages/monocytes in response to pro-inflammatory stimuli including bacterial lipopolysaccharide (LPS) and tissue necrosis factor-α (TNF-α), an acute inflammatory cytokine [32,33]. The S100/calgranulins, especially S100A12 and S100b, constitute a family of calcium-binding polypeptides observed in PMNs, monocytes, and lymphocytes [34]. When released at sites of inflammation, S100s amplify inflammatory responses by inducing adhesion molecule expression, cytokine secretion, and the release of tissue-destructive proteinases from phagocytes [35]. In contrast to short-lived cellular activation mediated by LPS, engagement of RAGE by AGEs, HMGB1, or S100s results in sustained inflammation [15].

Analysis of control upper airway respiratory mucosa revealed normal respiratory epithelium and diffuse glands; however, samples from patients with documented CS revealed pronounced goblet cell and submucosal gland hyperplasia, leukocyte extravasation, and RAGE immunoreactivity. These discoveries demonstrate for the first time that RAGE, a cell surface pattern recognition receptor implicated in tobacco smoke-induced disease is up-regulated in sinonasal tissues and upper airways. Accordingly, the functional contributions of RAGE signaling in the context of smoke exposure warrant investigation so that mechanistic understanding of CS progression can be clarified. 

## 2. Materials and Methods

### 2.1. Human Control and CS Samples

Formalin-fixed paraffin-embedded tissue samples from normal and CS patients were obtained from Precision for Medicine (Carlsbad, CA, USA). The human slides were representative of a male human population 58 ± 7.2 years of age. Further, human slides provided from Precision for Medicine used in the staining procedures outlined below were scored and confirmed by a board-certified pathologist with all appropriate human ethical approvals maintained by the entity.

### 2.2. Mice and Animal Use

Female wild-type (WT) mice (Jackson Laboratories; Bar Harbor, ME, USA) in a C57BL/6 background were supplied with food and water ad libitum in a specific pathogen free facility and maintained on a 12-h light–dark cycle. Because exposure to tobacco products can induce and/or exacerbate CS [36], we exposed mice (n = 8) to secondhand smoke (SHS; derived from 3R4F research cigarettes from the Kentucky Tobacco Research and Development Center, University of Kentucky using a nose-only exposure system) from 12 weeks of age until 18 weeks of age (InExpose System, Scireq, Montreal, QC, Canada) as previously described [37]. Mice that were similarly restrained but exposed to room air (RA) were used as controls. At the conclusion of the exposure, mice were sacrificed, and biopsies that contained sinus cavities were inflation-fixed with 4% paraformaldehyde for histology. 

As similarly performed previously by our lab [38], two transgenic lines of mice were mated to create conditional doxycycline (dox)-inducible mice that overexpress RAGE by sinus epithelium. Specifically, mice that harbor a Tet-On RAGE transgene [38] were mated to a mouse that contained the reverse tetracycline transactivator (rtTA) under the control of a Keratin-14 (K14) promoter as K14 is abundantly expressed by sinus epithelium [39]. K14-rtTA/TetO-RAGE mice (hereafter RAGE TG mice) were viable and phenotypically indistinguishable from control littermates. It is possible that use of the K14 promoter targets other non-sinus tissues; however, no sinus-specific promoters exist and we have not observed any evidence of epidermal abnormalities or skin inflammation in these RAGE TG mice to date. Female RAGE TG mice that contained both transgenes and single or non-transgenic controls (n = 8 per group) were fed dox (625 mg/kg; Harlan Teklad, Madison, WI, USA) for six consecutive weeks, from 12 to 18 weeks, then sacrificed. On the date of sacrifice, biopsies that contained sinus cavities were inflation-fixed with 4% paraformaldehyde for histology or were used to isolate total protein or RNA. Mice were housed and utilized in accordance with an animal use protocol (#150403) approved by the Institutional Animal Care and Use Committee (IACUC) at Brigham Young University and carried out in accordance with the prevailing regulations.

### 2.3. Histology

Slides of human sinus epithelium, obtained from normal or CS patients, were stained with hematoxylin and eosin (H&E, Thermo Scientific, Pittsburg, PA, USA) to observe general morphology. Immunohistochemical localization of RAGE (R&D Systems, Minneapolis, MN, USA; Cat #mAb1179, 1:500) or S100s (Sigma Aldrich, St. Louis, MO, USA; S2644, 40 mg/mL) was also performed on sections of human tissue as previously outlined. The images shown are representative of at least 4 images per human sample and the cohort included n = 8 per human group. For each immunohistochemical stain involving mouse samples, at least eight images were evaluated from each mouse (n = 8 per group). A no-primary antibody control was performed in each immunostaining experiment, and in each case, this negative control had no immunoreactivity. Stained sections were imaged using an Olympus BX51 microscope and Olympus CellSens Standard 3.1 (Olympus, Tokyo, Japan). Stained slides were evaluated for immunoreactivity using Image J (Version 1.54c, U.S. National Institutes of Health, Bethesda, MD, USA) densities in control samples were normalized to 1 and then compared to densities in other groups.

### 2.4. Tissue Isolation and Characterization

Sinonasal epithelium was removed from murine nasal septa and sinus cavities by dissection and washed with HEPES-DMEM containing 1% penicillin-streptomycin to remove blood and other debris. The resulting tissues were dissociated and prepared for protein isolation after the manner published by Davidson et al., 2004 [40]. Total RNA was isolated from resected tissue using Trizol reagent (Sigma, St. Louis, MO, USA). After total RNA was spectrophotometrically quantified, reverse transcription and PCR amplification was conducted with a One-Step Brilliant SYBR Green qRT-PCR Master Mix kit (Stratagene, San Diego, CA, USA) and an anMx3000P real-time PCR system computerized cycler from Stratagene. The following primers were synthesized and HPLC purified by Invitrogen Life Technologies (Carlsbad, CA, USA): MMP-9 (forward, ATG ACA GCT GCA CCA CTG AG; reverse, ATT TGT TGC CCA GGA AAG TG); MMP-13 (forward, GCC ACC TTC TTC TTG TTG AGT TG; reverse, GAC TTC TTC AGG ATT CCC GCA); and 18s RNA (forward, GTA ACC CGT TGA ACC CCA TT; reverse, CCA TCC AAT CGG TAG TAG CG). cDNA (10 ng) and primer pairs (75 nM each) were used in a total volume of 25 mL. Cycle parameters were as follows: 40 min at 55 °C for reverse transcription, followed by 10 min at 95 °C, and 40 cycles composed of 30 s at 95 °C, 1 min at 58 °C, and 30 s at 72 °C. Control wells lacking template or RT were included to identify primer–dimer products and to exclude possible contaminants. These experiments were performed in triplicate and n = 8 for each group.

Protein isolation was performed by homogenization with RIPA buffer containing protease inhibitors (Fisher Scientific, Waltham, MA, USA). Total protein was quantified using a BCA Protein Assay Kit (Fisher Scientific) and 20 mg of protein was used for active immunoblotting or characterization of cytokines. Protein lysates were separated by electrophoresis through Mini-PROTEAN TGX Precast gels (Bio-Rad Laboratories, Hercules, CA, USA) and transferred to nitrocellulose membranes. Membranes were incubated overnight with antibodies against active caspase-3 or β-Actin (Cell Signaling, Danvers, MA, USA). Membranes were then incubated with fluorescent secondary antibodies for an hour and washed ×3 with TBS-Tween the next day prior to imaging. Membranes were developed on a Li-COR Odyssey CLx (Li-COR Biosciences, Lincoln, NE, USA). Fluorescence densities were determined, and comparisons were made between mouse groups. These experiments were performed in triplicate and n = 8 for each group.

Mouse enzyme linked immunosorbent assay (ELISA) kits were used to quantify cytokines in freshly procured sinus protein samples. Briefly, equal concentrations of protein were assayed in each group (n = 8 mice per group) for concentrations of IL-1β (Cat #ELM-IL1β), Ray Biotech, Norcross, GA, USA), IL-3 (Cat # ELM-IL3, Ray Biotech) and TNF-α (Cat # ELM-TNFα, Ray Biotech) as directed by the manufacturers.

### 2.5. Statistical Analyses

Results were checked for normality, and data were shown as means ± SE. Differences were determined between control and experimental groups. Mann–Whitney tests were used to compare the changes in the protein or RNA expression. Statistical analysis was performed with GraphPad Prism 8.0 software, and significant differences were noted at *p* ≤ 0.05. 

## 3. Results

### 3.1. Characterization of Sinus Tissue from Human CS and Normal Patients

**Figure 1:**
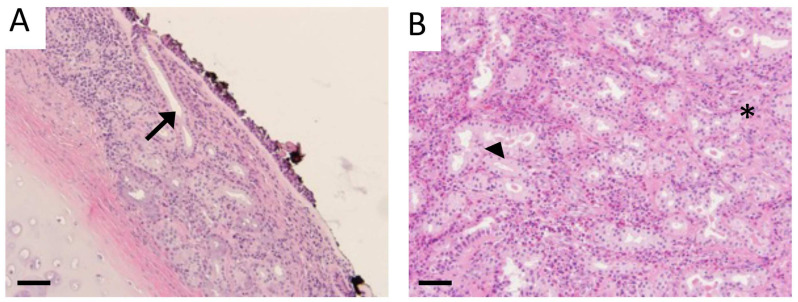
H&E staining of human tissue from CS patients (**B**) revealed hyperplasia of goblet cells and submucosal glands (*), stromal edema, and leukocyte extravasation (arrowhead) compared to normal control tissue (arrow, **A**). Images are representative of 4 randomized fields obtained from 8 samples from each group and scale bars are 50 mm.

**Figure 2:**
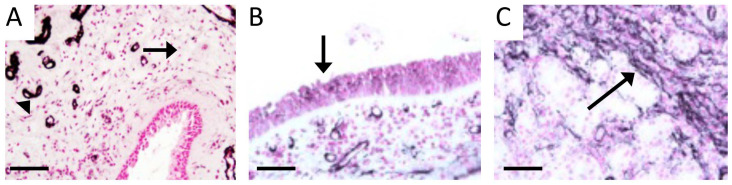
Immunohistochemistry for RAGE in normal human sinus tissue revealed only faint staining in submucosal glands (**A**, arrow) and intense staining of vasculature (**A**, arrowhead). In samples from CS patients, RAGE was intensely localized to respiratory epithelium (**B**, arrow) and basolateral edges of glandular epithelium in the mucosa (**C**, arrow). Images are representative of 4 randomized fields obtained from 8 samples from each group and scale bars are 50 mm.

**Figure 3:**
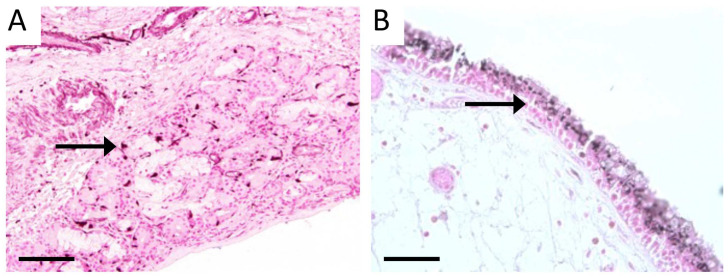
Immunostaining for S100s, common RAGE ligands, was not evident in control human respiratory epithelium but was apparent in myoepithelial cells that surround glandular ducts (**A**, arrow). There was intense expression of S100s by sinus epithelium in patients with CS (**B**, arrow). Images are representative of 4 randomized fields obtained from 8 samples from each group and scale bars are 50 mm.

Histological slides procured from a human tissue bank (Precision for Medicine) were confirmed from normal donors or those with CS. Staining of sections with a traditional H&E stain revealed marked goblet cell hyperplasia, particularly in submucosal glands, and notable stromal edema in CS tissues compared to normal controls (Figure 1). These observations delineated morphological alterations in diseased vs. normal tissue. Immunostaining of normal human sinus tissues with RAGE specific antibodies revealed faint immunoreactivity in submucosal glands deep to sinus epithelium (Figure 2C, arrow) compared to controls (Figure 2A, arrow). Interestingly, RAGE localization was elevated in normal vascular areas of normal tissue (Figure 2A). Sinus epithelium in CS samples expressed abundant RAGE protein (Figure 2B) in comparison to near-absent epithelial expression in normal samples (Figure 2A). Total RAGE immunoreactivity in control human samples was 1.0 ± 0.07 and 1.33 ± 0.12 in CS patients. RAGE binds diverse ligands and a key family of ligands include S100/calgranulins (S100s). We therefore sought to assess the expression of this ligand family known to initiate RAGE signaling. We discovered poor qualitative expression of S100s in normal sinus tissues (Figure 3A); however, expression of S100s was intensely detected in the epithelial lining of sinus cavities of CS patients (Figure 3B). Relative detection of S100s in human control samples was 1.0 ± 0.11 and 1.57 ± 0.11 in tissues from CS patients.

### 3.2. RAGE and Potential Mouse Models of CS

**Figure 4:**
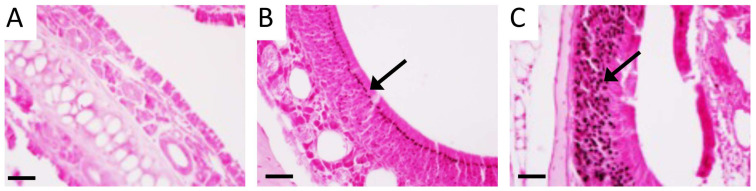
IHC showed undetectable RAGE expression in mouse sinus epithelium after exposure to room air (**A**) and punctate expression throughout the epithelial lining of sinuses following 4 weeks of SHS exposure (**B**, Arrow). RAGE expression was also increased in diffuse mucosal cells of the sinus following SHS exposure (**C**, Arrow). Images are representative of 8 randomized fields obtained from 8 samples from each group and scale bars are 50 mm.

**Figure 5:**
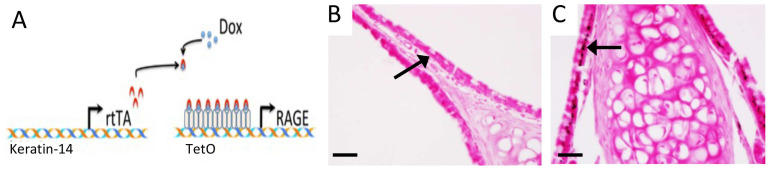
Model for producing conditional RAGE over-expressing transgenic mice (**A**). RAGE was undetected in non-transgenic controls (**B**, Arrow) and augmented in sinus epithelium from RAGE TG mice under the control of the Keratin-14 promoter (**C**, Arrow). Images are representative of 8 randomized fields obtained from 8 samples from each group and scale bars are 50 mm.

Because exposure to tobacco smoke causes and often exacerbates CS, we next sought to assess RAGE expression in sinus tissues following exposure to SHS. We did not observe RAGE expression in sinus epithelium isolated from control mice exposed to room air (Figure 4A). However, abundant RAGE expression was observed in sinus epithelium in mice following acute exposure to SHS for 6 weeks (Figure 4B). Moreover, RAGE was also elevated in mucosal cells following SHS exposure (Figure 4C). Total RAGE immunoreactivity in control mouse sinus tissue was 1.0 ± 0.13 and 1.87 ± 0.17 in sinus tissues from mice exposed to SHS. As RAGE expression in sinus tissue increases following SHS exposure (Figure 4), we next contemplated genetically inducing RAGE expression, in the absence of SHS, in order to model RAGE-mediated sinus compromise. Accordingly, we utilized a Tet-On mouse model in order to up-regulate RAGE expression in sinus cavities. We used a mouse that harbored an rtTA transgene downstream of the keratin-14 promoter and a separate line that contained a RAGE cDNA sequence under the control of TetO response elements (Figure 5A). Double transgenic mice that contain both transgenes (RAGE TG) expressed RAGE when fed dox. RAGE was not detected in sinus epithelium in non-transgenic controls (Figure 5B), which was similar to the lack of RAGE expression in room air control mice (Figure 4A). RAGE TG mice fed dox for 6 weeks experienced abundant RAGE expression in sinus epithelium observed via immunohistochemical staining for RAGE (Figure 5C, arrow). 

**Figure 6:**
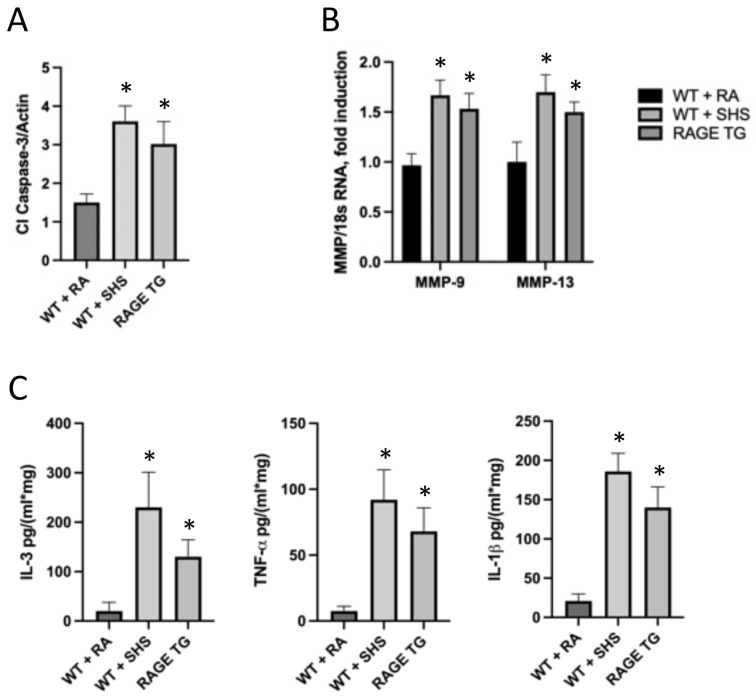
Active caspase-3 isoforms were increased in SHS-exposed WT and RAGE TG mice compared to controls (**A**). MMP mRNA transcripts were significantly elevated in SHS-exposed and RAGE TG mice compared to room air controls (**B**). IL-3, TNF-α and IL-1β were significantly elevated in SHS-exposed WT and RAGE TG mice compared to controls (**C**). * in the figure indicates a significant difference.

RAGE TG mice were also screened for markers of inflammation and remodeling and compared to WT mice exposed to room air or SHS (Figure 6). We discovered that sinus tissue from SHS-exposed mice expressed significantly more cleaved caspase-3 (Cl Caspase-3) compared to RA controls (Figure 6A) suggesting apoptosis is a cellular response to exposure. Furthermore, 6 weeks of RAGE up-regulation in RAGE TG mice expressed a similarly significant increase in Cl Caspase-3 (Figure 6A) revealing RAGE expression is sufficient to initiate this apoptotic pathway. Matrix metalloproteases (MMPS) are key regulators of cell growth, invasion, and angiogenesis [41]. Evaluation of tissue remodeling enzymes MMP-9 and -13 resulted in elevated transcription of these two genes in mice exposed to SHS or in RAGE TG mice (Figure 6B). In addition to markers that control cell turnover (Cl Caspase-3) and tissue remodeling (MMPs), we evaluated the expression of pro-inflammatory cytokines that function during immune responses. We observed anticipated increases in the expression of IL-3, TNF-α, and IL-1β in mouse sinus tissue following exposure to SHS (Figure 6C). Interestingly, we observed a significant increase in the synthesis and secretion of these same inflammatory mediators when RAGE was genetically augmented in RAGE TG mice (Figure 6C).

## 4. Discussion

Sinus cavities are composed of diverse tissue types including surface epithelium, highly vascularized mucosa, and glandular structures. Sinus cavities are specifically lined with pseudostratified ciliated columnar epithelial cells interspersed with mucous goblet cells so that cilia continuously sweep the mucous toward ostial openings. Their anatomical interposition between the organism and the environment make them particularly prone to damage and various pathological responses. The current research demonstrated that patients diagnosed with CS express excessive mucus-secreting goblet cells and expanded submucosal glands compared to normal patients. CS causes notable morbidity due to numerous and diverse causative factors. Further, many have postulated that both primary and SHS correlate with more severe symptoms of CS [36]. For instance, studies such as prospective cross-sectional endeavors have shown that elevated cotinine, a byproduct of nicotine metabolism during tobacco smoke exposure, correlated with exacerbations and overall quality of life [36]. Our discovery that SHS exposure in the mouse culminated in CS characteristics in sinus tissues buttress the observation that CS symptoms are highly common both in smokers and in patients with COPD [42]. Furthermore, exposure to SHS, both during childhood and adulthood, posed a significant risk factor for CS [43]. A recent publication by Kuhar et al. evaluated the histopathologic consequences of never smokers, former smokers, and current smokers and quantitatively discovered smoking status related to hyperplastic changes, metaplasia, and fibrosis [44]. The current investigation provides further support for new research into the pathology of CS in the context of tobacco smoke exposure.

We have previously confirmed that exposure to primary and SHS leads to elevated RAGE expression [37,45], although the specific substances in tobacco smoke that directly up-regulate RAGE are not well characterized. Discoveries confirming differential RAGE expression in multiple tissue types following exposure suggest RAGE performs a central role in cellular responses to tobacco or other similar stimuli. Confirmation of mechanisms stemming from receptor augmentation in sinus tissues was further solidified by the observation of elevated S100s. S100s represent a ligand type that perpetuates RAGE-mediated signaling [46]. Differential release of S100s was initially published by Van Crombruggen et al., who described S100 release during upper airway inflammatory events [47]. They highlighted functional roles for these molecules that signal inflammatory mediators via pattern recognition receptors. HMGB-1 is another parallel RAGE ligand that signals through the HMGB1-AGE-NF-kB axis [48] that has been identified previously in sinus tissues that progress to CS [49,50]. In the future, the evaluation of AGEs, a set of diverse RAGE ligands, should also be assessed in order to further develop the narrative that RAGE functions in SHS-induced CS. AGEs are reactive, cross-linked entities that form via reactions between reducing sugars and amino groups that comprise proteins, lipids, and nucleic acids. In fact, while no particular AGE dominates, myriad are formed in tobacco smoke [51]. While these seminal observations do not prove SHS exposure leads to CS, these data suggest that smoke exposure is associated with RAGE expression and that RAGE in sinus cavities may coincide with CS. Subsequent research that involves RAGE abrogation by genetic targeting may clarify plausible roles for RAGE in CS.

We recognize that our newly developed K14-rtTA/TetO-RAGE mouse described in this publication is not a perfect mouse model for CS. Despite expression of K14 by sinus epithelium [39], there is a high likelihood that off-target RAGE up-regulation (due to the expression pattern of K14) is possible. Additional research is required that exhaustively characterizes this mouse in terms of inflammatory mediator expression, proliferation/apoptosis of specific cell populations, and complete histopathology. However, we demonstrate that this mouse model in fact up-regulates RAGE expression without the stimulus of tobacco smoke exposure and many CS-related changes result. For instance, we discovered that cleaved caspase-3 was increased following either SHS exposure or RAGE up-regulation. Such an observation is supported by similar findings by others that sought to clarify this factor involved in mitochondrial-mediated mechanisms of apoptosis [52,53]. MMPs are proteases with central roles in cell growth, invasion, and angiogenesis [41]. MMP-9 is a protease that functions in many biological processes and has been widely associated with the pathology of CS growth [54,55]. MMP-13 is an additional protease that tends to be expressed during pathological inflammatory events and is particularly elevated by sinus epithelium experiencing remodeling as the disease progresses [56,57]. We were therefore intrigued by our observation that the transcription of these genes were significantly elevated by SHS exposure, but similarly by RAGE TG mice. 

We observed that pro-inflammatory cytokines classically associated with tobacco-mediated CS were elevated by RAGE TG mice. IL-1β and TNF-α play a major role in the progression of acute sinusitis and that IL-3 dominates the cytokine profile in chronic sinusitis—leading to the coordinating effects of a variety of inflammatory cells [58,59]. IL-3 regulates the biology of progenitor cells and activates neutrophils and macrophages. TNF-α modulates the expression of myriad cytokines during inflammation, participates in leukocyte diapedesis, and is highly expressed in CS patients [58]. Similarly, IL-1β is expressed in inflammatory conditions and it often partners with TNF-a in the coordination of cytokine synthesis and secretion as well as during leukocyte chemotaxis [60]. 

In summary, our work demonstrated that SHS can induce inflammation and other CS phenotypes in mice and that RAGE up-regulation was sufficient to similarly induce these CS-related characteristics. This study provides an important step in dissecting possible roles for RAGE and its coordinated mechanisms that may orchestrate CS pathology.

## Data Availability

All data are presented within the article. Data and other materials are available from the corresponding author on reasonable request.
